# The association between global and prime diet quality scores and the risk of breast cancer: a case control study

**DOI:** 10.3389/fpubh.2026.1756331

**Published:** 2026-02-16

**Authors:** Hassan M. Otifi, Georgios Zacharakis, Saleh Hussain A. Almasabi, Khalid Alhazmi, Nahla Kambal, Bandar Alshreef, Ahlam Saleh Alhajri

**Affiliations:** 1 Pathology Department, College of Medicine, King Khalid University, Abha, Saudi Arabia; 2 Department of Internal Medicine, College of Medicine, Prince Sattam bin Abdulaziz University, Al-Kharj, Saudi Arabia; 3 Department of Clinical Laboratory Sciences, College of Applied Medical Sciences, Najran University, Najran, Saudi Arabia; 4 Department of Pathology, University of Tabuk, Tabuk, Saudi Arabia; 5 Department of Clinical Nutrition, College of Nursing and Health Sciences, Jazan University, Jazan, Saudi Arabia; 6 Department Medical Laboratory, Shaqra University, Shaqra, Saudi Arabia; 7 Department of Food Science and Nutrition, College of Agricultural and Food Sciences, King Faisal University, Al-Ahsa, Saudi Arabia

**Keywords:** breast cancer, GDQS, global diet quality, PDQS, prime diet quality

## Abstract

**Backgrounds:**

Despite established links between diet and breast cancer, the association of comprehensive diet quality indices specifically Global Diet Quality Score (GDQS) and Prime Diet Quality Score (PDQS) with BC risk remains underexplored. This case-control study evaluated the association between global and prime diet quality scores and the risk of breast cancer.

**Methods:**

Dietary intake of 125 newly diagnosed, histopathologically confirmed breast cancer cases and 250 age–frequency–matched controls (women ≥30 years) at Shaqra General Hospital was assessed using a validated semi-quantitative FFQ, and diet quality was evaluated by GDQS and PDQS indices, with multivariable logistic regression models (SPSS v26) adjusted for age, BMI, total energy intake, physical activity, family history of breast cancer, and smoking status, including trend analyses across tertiles of diet quality scores.

**Results:**

Breast cancer cases had significantly lower total GDQS (*p* = 0.002) and total PDQS (*p* < 0.001) than controls. In multivariable models, higher total GDQS and positive GDQS scores were strongly associated with lower odds of breast cancer (e.g., total GDQS highest tertile aOR = 0.397; 95% CI: 0.279–0.653; Ptrend < 0.001), and higher total PDQS scores were similarly protective (aOR = 0.455; 95% CI: 0.309–0.761; Ptrend = 0.003). Negative GDQS and unhealthy PDQS scores were not significantly associated with breast cancer risk.

**Conclusion:**

A high diet quality, as indicated by high total GDQS and total PDQS scores, was associated with a decreased risk of breast cancer. These findings highlight a potential role of overall diet quality in breast cancer etiology but should be interpreted as observational associations rather than evidence of causal or therapeutic effects.

## Introduction

Breast cancer remains the most prevalent cancer among women worldwide, with approximately 2.3 million new cases diagnosed in 2020, accounting for about 12% of all cancer cases (1). In Saudi Arabia, the incidence of breast cancer has been alarmingly increasing, with 3,954 new cases reported in 2020, making it the most commonly diagnosed cancer among women in the country (2). The age-standardized incidence rate (ASIR) reached 28.8 cases per 100,000 population in 2020, reflecting a significant public health challenge (3). The rising incidence, particularly in low- and middle-income countries, underscores the urgent need to identify modifiable risk factors for prevention. Diet, a critical modifiable factor, influences cancer risk through mechanisms such as inflammation, oxidative stress, and hormonal regulation (4). Diet quality scores, which evaluate adherence to healthy dietary patterns, provide a comprehensive approach to studying these effects compared to single-nutrient analyses.

The global burden of breast cancer stems from both non-modifiable factors, such as genetics, and modifiable factors, like lifestyle (5). While genetic mutations (e.g., BRCA1/2) account for 5%–10% of cases, most breast cancers are sporadic, suggesting environmental influences (6). Early dietary research focused on nutrients like fats or antioxidants, but inconsistent findings—such as null associations between saturated fat and breast cancer risk shifted focus to dietary patterns (7). Diet quality indices, such as the Healthy Eating Index (HEI-2015), Mediterranean Diet Score (MDS), and Diet Quality Index-International (DQI-I), evaluate overall diet based on fruits, vegetables, whole grains, and limited processed foods (8). These scores, adaptable to regional diets, are linked to reduced chronic disease risk, including breast cancer, especially in postmenopausal women (9).

Physiologically, high-quality diets provide bioactive compounds like polyphenols and fiber, which reduce inflammation and oxidative stress, key carcinogenic drivers (10). Fiber lowers circulating estrogens, potentially reducing hormone receptor-positive breast cancer risk (11). Conversely, prime quality diets high in processed meats and sugars promote inflammation and insulin resistance, increasing risk (12).

Research on diet quality and breast cancer risk shows varied findings, particularly when considering menopausal status and tumor subtypes. The GDQS, a relatively new tool, evaluates overall dietary patterns by emphasizing nutrient dense foods, such as fruits, vegetables, and lean proteins, and penalizing harmful components like processed items and excessive fats (13). A study by Bromage et al. (14) introduced the GDQS, highlighting its utility in assessing diet quality across diverse populations. Similarly, the PDQS focuses on optimal eating patterns for chronic disease prevention, prioritizing whole foods and limiting refined sugars and unhealthy fats (15, 16).

This case-control study aims to investigate the association between global and prime diet quality scores and the risk of breast cancer.

## Materials and methods

### Study population

This case–control study was conducted at Shaqra General Hospital and specifically included female participants aged 30 years or older. Data collection was carried out from April 2023 to March 2025. The case group comprised 125 women with histopathologically confirmed breast cancer. To ensure a relatively homogeneous case group, we applied several exclusion criteria: women with any history of other cancers, those currently receiving hormone replacement therapy (HRT), pregnant or breastfeeding women, and individuals following specific dietary regimens (such as vegetarian diets) were excluded from participation.

For the control group, we selected 250 women from the same hospital source population who were being treated for various non-neoplastic conditions unrelated to known breast cancer risk factors during the same period (April 2023 to March 2025). Controls were frequency-matched to cases by age in an approximately 1:2 ratio to achieve a similar age distribution between groups. The control selection process used random sampling from eligible women attending Shaqra General Hospital outpatient and inpatient services, rather than aiming for population representativeness. For controls, additional exclusion criteria included a diagnosis of any chronic metabolic or gastrointestinal condition known to necessitate major, long-term dietary modifications. This specifically included: type 1 or type 2 diabetes requiring insulin or strict dietary therapy, inflammatory bowel disease (Crohn’s disease or ulcerative colitis), celiac disease, chronic kidney disease (stage 3 or higher), and severe cardiovascular diseases (e.g., recent myocardial infarction or stroke) under specific therapeutic diets. These conditions were selected *a priori* because their management often requires structured dietary interventions that could substantially alter habitual diet quality scores, making them non-representative of the general population’s dietary habits unrelated to disease management.

All participants underwent rigorous screening to confirm they met the study criteria. The comprehensive exclusion criteria applied to both cases and controls included any history of cancer (regardless of site), current HRT use, pregnancy or lactation status, and adherence to specialized dietary practices. For controls, we additionally excluded those with benign breast disease or conditions potentially related to breast cancer development. The two groups were similar in age, and the final study population included a total of 375 participants (125 cases and 250 controls) who met all inclusion criteria without protocol violations. This carefully designed frequency-matching strategy and thorough exclusion process helped minimize potential confounding factors while maintaining sufficient sample size for robust statistical analysis. The study design particularly focused on newly diagnosed, untreated breast cancer cases to better assess potential dietary risk factors without the confounding effects of cancer treatment.

### Dietary assessment

Participants’ dietary habits were assessed through a validated semi-quantitative Food Frequency Questionnaire (FFQ) consisting of 152 food items commonly consumed within the local community (17). This instrument was designed to capture usual dietary intake over the past 12 months. Each participant was instructed to report the frequency of consumption for each listed food item by choosing from a standardized set of nine response categories. These categories ranged from “never or less than once per month” to “six or more times per day,” allowing for a nuanced quantification of intake across a broad spectrum of consumption behaviors. To improve the accuracy of self-reported data, respondents were provided with household measurement units familiar to them—such as cups, tablespoons, teaspoons, slices, or pieces—as well as visual aids, including color photographs depicting portion sizes. These tools were instrumental in helping participants more accurately estimate the typical amount of each food they consumed. Following data collection, the reported consumption frequencies were converted into average daily intake values, expressed in grams. Subsequently, the dietary intake data were entered and analyzed using Nutritionist IV software, a comprehensive dietary analysis program that incorporates an extensive food composition database (18). This database includes detailed information on the nutrient content of foods, allowing for the precise estimation of each participant’s daily intake of total energy, macronutrients (carbohydrates, proteins, and fats), micronutrients (vitamins and minerals), and various bioactive food components such as fiber, flavonoids, and antioxidants. These calculations provided the foundation for further statistical analyses examining the relationship between dietary intake and various health outcomes in the study population (18).

### Global diet quality score (GDQS)

The Global Diet Quality Score (GDQS) was calculated by categorizing each participant’s daily food intake (measured in grams) into 25 separate food groups. These groups included 16 categories recognized for their positive health effects (such as fish and shellfish, poultry and game meats, eggs, low-fat dairy, whole grains, cruciferous vegetables, dark green leafy vegetables, deep orange vegetables, other vegetables, citrus fruits, deep orange fruits, other fruits, deep orange tubers, legumes, nuts and seeds, and liquid oils), two groups with moderate health benefits (red meat and high-fat dairy), and seven groups identified as potentially harmful (including refined grains and baked goods, white roots and tubers, fruit juices, sugar-sweetened beverages, sweets and ice creams, fried foods, and processed meats). Each food group was further divided into three or four levels of consumption.

The scoring system for the health-enhancing groups was as follows:

- A score of 0 was given for low consumption in all 16 beneficial categories.
- For cruciferous vegetables, deep orange vegetables, other vegetables, and deep orange tubers, moderate intake received 0.25 points and high intake 0.5 points.
- Citrus fruits, deep orange fruits, other fruits, whole grains, liquid oils, fish and shellfish, poultry and game meats, and low-fat dairy were assigned 1 point for moderate and 2 points for high consumption.
- Eggs, dark green leafy vegetables, deep orange vegetables, legumes, and nuts and seeds were scored 2 points for moderate and 4 points for high intake.

For the two moderately beneficial groups:

- Both low and very high intakes were assigned 0 points.
- Moderate intake was scored as 1 point.
- High consumption received 2 points for both red meat and high-fat dairy.

For the seven less healthy food groups:

- Low intake was rewarded with 2 points.
- Moderate consumption was given 1 point.
- High intake was scored 0 for refined grains, white roots and tubers, fruit juices, sugar-sweetened beverages, sweets and ice creams, fried foods, and processed meats.

The overall GDQS was determined by adding the points from all 25 food groups, resulting in a possible score between 0 and 49 (14).

### Prime diet quality score (PDQS)

The Prime Diet Quality Score (PDQS) is based on the intake of 21 distinct food groups, which are classified into two categories: beneficial (healthy) and detrimental (unhealthy) dietary components. Each of these food groups was initially categorized into tertiles based on consumption levels. For the healthy components such as low-fat dairy products, poultry, whole grains, fish and shellfish, legumes and soy products, nuts and seeds, vegetable oils, citrus fruits, other fruits, deep orange fruits, cruciferous vegetables, dark leafy greens, deep orange vegetables, and other vegetables participants were awarded scores as follows: 0 points for the lowest tertile, 1 point for the middle tertile, and 2 points for the highest tertile of intake. In contrast, for unhealthy dietary components such as processed meats, red meats, sugar-sweetened beverages, refined grains, fried foods, and sweets the scoring system was reversed: participants received 2 points for the lowest tertile, 1 point for the middle tertile, and 0 points for the highest tertile. The overall PDQS score, derived by summing the scores of all food groups, ranges from 0 to 42, with higher scores reflecting better diet quality (19).

### Statistical analysis

All data analyses were performed using IBM SPSS Statistics software, version 26.0 (IBM Corp., Armonk, NY, USA). Categorical variables were analyzed using the Chi-square test to compare proportions between groups. For continuous variables, either the independent samples t-test or the Mann–Whitney U test was employed based on the normality of the data distribution. Results for continuous variables are presented as mean ± standard deviation (SD) or median with interquartile range (25th–75th percentile), as appropriate, while categorical variables are expressed as percentages.

Because controls were frequency-matched to cases by age rather than individually matched, unconditional binary logistic regression models were used to examine the associations of GDQS and PDQS with breast cancer risk. Age was included as a covariate in all multivariable models, along with other potential confounders. Trend analyses across tertiles of diet quality scores were also conducted by entering the median value of each tertile as a continuous variable. Both crude (unadjusted) and adjusted models were constructed, and findings are presented as odds ratios (ORs) with corresponding 95% confidence intervals (CIs).

## Results

Table 1 summarizes the baseline characteristics and dietary intakes of participants in the breast cancer (*n* = 125) and control (*n* = 250) groups. There were no significant differences between the two groups in terms of age, BMI, physical activity, marital status, or smoking history (*p* > 0.05 for all). However, a significantly higher proportion of breast cancer patients reported a family history of breast cancer compared to controls (11.2% vs. 3.6%, *p* = 0.026). There were no significant differences in energy or carbohydrate intake between the groups. However, the breast cancer group had lower protein (72.2 ± 13.2 vs. 83.1 ± 14.8 g/day; *p* = 0.024) and fiber intake (16.8 ± 2.4 vs. 20.8 ± 3.2 g/day; *p* = 0.037), but higher fat intake (68.1 ± 8.3 vs. 58.2 ± 8.1 g/day; *p* = 0.041) compared to controls.

**Table 1:** Comparison of baseline characteristics between healthy individuals and breast cancer patients.

Variable	Breast cancer (*n* = 125)	Control (*n* = 250)	*p*-value
Baseline characteristics			
Age (year)^a^	56.5 ± 9.1	56.9 ± 9.2	0.425
BMI (kg/m^2^)^a^	27.1 ± 3.9	26.3 ± 3.4	0.297
Physical activity (MET/h/day)^a^	38.2 ± 3.4	38.7 ± 3.6	0.851
Familial history of breast cancer, yes, %^b^	12 (11.2%)	9 (3.6%)	**0.026**
Marital status (married) *n* (%)^b^	109 (87.2)	216 (86.4)	0.412
Smoking history, yes, %^b^	11 (8.8%)	13 (5.2%)	0.228
Dietary intakes			
Energy (kcal/day)^a^	2362.3 ± 392.3	2248.1 ± 348.2	0.642
Carbohydrate (g/day)^a^	354.4 ± 74.2	340.7 ± 70.8	0.367
Protein (g/day)^a^	72.2 ± 13.2	83.1 ± 14.8	**0.024**
Fat (g/day)^a^	68.1 ± 8.3	58.2 ± 8.1	**0.041**
Fiber (g/day)^a^	16.8 ± 2.4	20.8 ± 3.2	**0.037**

BMI, body mass index; MET, metabolic equivalent of task.

Values are mean ± SD for continuous and frequency (percentage) for categorical variables.

a Using independent samples T-test for normal continuous variables.

b Using chi-square test for categorical variables.

Significant values (*p* < 0.05) are presented in bold.

The results of Table 2 comparing food group intake and diet quality indices between 125 breast cancer patients and 250 healthy controls revealed statistically significant differences in dietary patterns. Breast cancer patients had a significantly lower total GDQS (*p* = 0.002) and total PDQS (*p* < 0.001), indicating a greater deviation from optimal dietary patterns. In terms of food consumption, patients consumed significantly lower amounts of protective foods, including dark leafy vegetables (*p* = 0.002), other vegetables (*p* = 0.004), other fruits (*p* = 0.041), legumes (*p* = 0.021), fish and shellfish (*p* = 0.003), and poultry (*p* = 0.040). In contrast, intake of refined grains and baked products (*p* = 0.003), sugar-sweetened beverages (*p* < 0.001), fried foods (*p* = 0.003), and sweets/ice cream (*p* = 0.002) was significantly higher among patients. No significant differences were observed for citrus fruits, orange-colored fruits, cruciferous vegetables, nuts, whole grains, oils, red meat, processed meats, eggs, or dairy products.

**Table 2:** Comparison of food group intake between healthy individuals and breast cancer patients.

Variable	Breast cancer (*n* = 125)	Control (*n* = 250)	*p*-value
Positive GDQS^a^	20.54 ± 3.25	22.89 ± 4.41	0.021
Negative GDQS^a^	15.81 ± 1.63	16.64 ± 1.64	0.033
Total GDQS^a^	30.17 ± 5.12	33.36 ± 5.42	0.002
Healthy PDQS^a^	18.99 ± 4.50	21.03 ± 4.94	0.041
Unhealthy PDQS^a^	14.76 ± 3.24	19.09 ± 4.53	0.005
Total PDQS^a^	26.22 ± 5.21	29.62 ± 6.36	<0.001
Citrus fruits (g/day)^b^	66.17 (26.25–80.95)	59.50 (12.94–112.92)	0.214
Deep orange fruits (g/day)^b^	78.46 (19.29–164.72)	80.42 (50.57–164.72)	0.358
Other fruits (g/day)^b^	62.27 (35.66–105.65)	89.53 (46.77–165.51)	0.041
Deep dark and leafy vegetables (g/day)^b^	31.52 (17.92–51.68)	51.69 (27.37–88.67)	0.002
Cruciferous vegetables (g/day)^b^	5.44 (6.92–95.27)	10.09 (6.83–18.75)	0.369
Deep orange vegetables (g/day)^b^	9.86 (6.52–16.17)	8.84 (6.07–16.21)	0.124
Other vegetables (g/day)^b^	162.34 (115.84–242.79)	210.05 (124.77–314.83)	0.004
Deep orange tubers (g/day)^b^	11.03 (5.70–17.69)	13.47 (9.40–19.34)	0.032
Legumes (g/day)^b^	24.81 (13.58–26.76)	35.96 (18.26–76.81)	0.021
Nuts and seeds (g/day)^b^	12.14 (6.88–16.72)	12.91 (7.88–13.91)	0.698
Whole grains (g/day)^b^	8.31 (6.89–10.83)	7.23 (6.33–6.34)	0.851
Refined grains and baked goods (g/day)^b^	428.17 (295.27–611.11)	342.41 (245.83–480.73)	0.003
White roots and tubers (g/day)^b^	20.42 (11.87–42.81)	31.19 (5.73–42.81)	0.512
Liquid oils (g/day)^b^	17.67 (13.21–25.97)	18.17 (15.64–24.97)	0.361
Red meats (g/day)^b^	50.75 (26.75–64.52)	52.43 (27.83–66.62)	0.210
Processed meats (g/day)^b^	8.18 (6.17–10.17)	6.88 (6.17–10.17)	0.254
Fish and shellfish (g/day)^b^	9.12 (6.87–14.83)	11.68 (7.69–20.24)	0.003
Poultry and game meats (g/day)^b^	18.78 (9.78–22.44)	24.31 (16.77–28.69)	0.040
Eggs (g/day)^b^	28.59 (14.59–34.34)	29.09 (14.73–34.34)	0.299
Low fat dairy products (g/day)^b^	230.64 (92.72–231.75)	252.84 (83.51–452.59)	0.574
High fat dairy products (g/day)^b^	64.45 (14.86–91.23)	65.91 (16.97–86.18)	0.264
Sweets and ice creams (g/day)^b^	15.66 (10.66–24.52)	44.59 (27.58–76.17)	0.002
Sugar-sweetened beverages (g/day)^b^	30.66 (10.88–60.88)	15.33 (8.70–40.71)	<0.001
Juices (g/day)^b^	8.88 (6.17–24.00)	9.56 (6.17–25.73)	0.421
Fried foods (g/day)^b^	20.17 (6.17–23.17)	15.07 (6.17–17.67)	0.003

GDQ, global diet quality; PDQ, prime diet quality.

a Using an independent sample *t*-test, values are presented as mean ± SD.

b Using the Mann–Whitney U-test, values are presented as median (25th–75th).

Table 3 presents an analysis of the relationship between diet quality scores and breast cancer risk, revealing significant inverse associations. After adjustment for age, BMI, physical activity, energy and nutrient intake, family history, and smoking, higher total GDQS demonstrated a strong protective effect. Participants in the highest tertile (T3) of total GDQS had substantially lower risk of breast cancer compared to the lowest tertile (T1) [Adjusted OR (aOR) = 0.397; 95% CI: 0.279–0.653; P-trend <0.001]. Similarly, higher Positive GDQS (reflecting healthy components) were associated with significantly reduced risk in T3 (aOR = 0.413; 95% CI: 0.281–0.711; P-trend <0.001). A significant protective trend was also observed for higher total PDQS (aOR for T3 = 0.455; 95% CI: 0.309–0.761; P-trend = 0.003). In contrast, Negative GDQS and Unhealthy PDQS showed no statistically significant association with risk after adjustment (P-trend = 0.263 and P-trend = 0.374, respectively). Healthy PDQS exhibited a non-linear relationship, with only the highest tertile (T3) showing significant risk reduction versus T1 (aOR = 0.575; 95% CI: 0.363–0.932; P-trend = 0.040).

**Table 3:** Relationship between tertiles of global and prime diet quality scores and breast cancer risk.

Variables	T₁	T₂ OR (CI 95%)	T₃ OR (CI 95%)	P_trend
GDQS				
Crude	Ref.	0.77 (0.51–1.232)	0.424 (0.314–0.622)	**0.004**
Adjusted	Ref.	0.675 (0.418–1.207)	0.397 (0.279–0.653)	**<0.001**
Positive GDQS				
Crude	Ref.	0.91 (0.596–1.46)	0.545 (0.383–0.838)	**<0.001**
Adjusted	Ref.	0.671 (0.416–1.196)	0.413 (0.281–0.711)	**<0.001**
Negative GDQS				
Crude	Ref.	1.059 (0.656–1.8)	0.61 (0.427–0.928)	**0.042**
Adjusted	Ref.	1.364 (0.738–2.691)	0.728 (0.43–1.38)	0.263
PDQS				
Crude	Ref.	0.719 (0.485–1.132)	0.415 (0.306–0.615)	**0.002**
Adjusted	Ref.	0.779 (0.478–1.38)	0.455 (0.309–0.761)	**0.003**
Healthy PDQS				
Crude	Ref.	1.478 (0.91–2.488)	0.635 (0.439–0.979)	**0.029**
Adjusted	Ref.	1.391 (0.763–2.693)	0.575 (0.363–0.932)	**0.040**
Unhealthy PDQS				
Crude	Ref.	0.838 (0.553–1.342)	0.687 (0.467–0.875)	**0.014**
Adjusted	Ref.	1.098 (0.616–1.81)	0.851 (0.454–1.822)	0.374

OR, risk ratio; CI, confidence interval; GDQS, global diet quality score, PDQS, prime diet quality score.

Adjusted model: adjusted for age (years), BMI (kg/m^2^), physical activity (MET/h/day), energy intake (kcal/day), Protein (g/day), fat intake (g/day), fiber intake (g/day), familial history of breast cancer (yes/no), and smoking history (yes/no).

Significant values (*p* < 0.05) are presented in bold.

## Discussion

This case-control study conducted at Shaqra General Hospital provides critical insights into the association between diet quality, assessed using the GDQS and PDQS, and breast cancer risk among women aged 30 years or older. The findings demonstrate that higher adherence to both GDQS and PDQS significantly reduces the risk of breast cancer.

The significant differences in dietary patterns between cases and controls underscore the protective role of specific food groups. Controls consumed higher amounts of fruits, dark green leafy vegetables, legumes, and poultry, while cases reported greater intake of refined grains, processed meats, sugar-sweetened beverages, and fried foods. These patterns are consistent with studies linking Plant-based, nutrient-rich diets can reduce breast cancer risk by decreasing exposure to carcinogenic compounds and increasing intake of beneficial phytochemicals and fiber (20). A 2013 study found that diets high in vegetables and low in processed foods were associated with a 15% lower breast cancer risk, supporting the current findings (21). The significant ORs for favorable GDQS components (OR = 0.235; 95% CI: 0.103–0.533) and healthy PDQS sub-scores (OR = 0.397; 95% CI: 0.185–0.854) indicate that nutrient-dense foods are the primary drivers of protection, though reduced consumption of detrimental foods also contributes (unfavorable GDQS: OR = 0.432; 95% CI: 0.249–0.750). The higher dietary fiber intake in controls aligns with evidence that fiber-rich foods, such as legumes and vegetables, lower breast cancer risk by reducing circulating estrogen levels, a key factor in hormone receptor-positive tumors (22). Conversely, the elevated fat intake in cases, particularly from processed sources, supports findings linking high-fat diets to increased tumorigenesis through metabolic dysregulation and chronic inflammation (23).

Physiologically, high-quality diets mitigate inflammation and oxidative stress, both implicated in breast cancer development (24). Fiber, abundant in the control group’s diet, reduces estrogen levels by binding bile acids and promoting fecal excretion, potentially lowering hormone-driven breast cancer risk (25). Lower intake of processed meats and sugar-sweetened beverages in controls may also decrease insulin resistance and pro-inflammatory cytokines, which are linked to elevated breast cancer risk (26). A 2021 study reported that high-fiber diets reduced inflammatory markers like C-reactive protein in women, supporting these mechanisms (27). The consistency of our findings with these studies suggests that GDQS and PDQS effectively capture dietary components influencing breast cancer risk through anti-inflammatory and hormonal pathways, offering a comprehensive approach to dietary assessment compared to single-nutrient analyses.

The public health implications of these findings are substantial, particularly in the context of rising breast cancer incidence in low- and middle-income countries (28). The strong inverse association between high-quality diets and breast cancer risk supports the promotion of dietary patterns rich in fruits, vegetables, legumes, and lean proteins. These findings align with global dietary guidelines, such as those from the EAT-Lancet Commission, which advocate plant-based diets for chronic disease prevention (8). The adaptability of GDQS and PDQS to local food systems makes them practical tools for tailoring interventions in diverse settings. For example, incorporating affordable legumes and locally available vegetables into dietary recommendations could enhance adherence in resource-constrained regions (29).

The significant differences in fat and fiber intake between cases and controls highlight specific dietary targets for intervention. The higher fat intake in cases aligns with evidence linking saturated fats to increased breast cancer risk through metabolic pathways (30), while the higher fiber intake in controls supports its protective role via estrogen modulation.

To further elucidate these findings, exploring the role of specific micronutrients within GDQS and PDQS frameworks is crucial. For instance, antioxidants like vitamins C and E, prevalent in fruits and vegetables, may neutralize free radicals, reducing DNA damage and subsequent cancer risk (31). In 2016, Cadeau et al. (32) founds that higher vitamin C intake was associated with a 12% lower breast cancer risk, particularly in postmenopausal women. Similarly, omega-3 fatty acids, found in fish and nuts consumed more by controls, may inhibit tumor growth by modulating cell signaling pathways (33). A 2024 study by Zhang et al. (34) reported a 10%–15% risk reduction with higher omega-3 intake, reinforcing the protective potential of these nutrients. Socioeconomic and cultural factors also warrant consideration, as access to nutrient-dense foods varies widely. In low-income settings, cost and availability may limit adherence to high GDQS and PDQS diets, a challenge noted in a study by Van Horn (35).

This study has several strengths. First, we used globally recognized and validated dietary indices (Global Diet Quality Score and Prime Diet Quality Score), which enhance the reliability and comparability of our findings. Second, dietary intake was assessed through face-to-face interviews using a semi-quantitative Food Frequency Questionnaire (FFQ), reducing the likelihood of misreporting. Third, the inclusion and exclusion criteria for both cases and controls were clearly defined, which improves internal validity. However, there are some limitations to consider. The case–control design is inherently prone to recall and selection biases and limits causal inference. Although efforts were made to enhance accuracy, dietary data were self-reported and may be subject to recall bias. Its single-center design, although conducted at a major referral hospital, may limit the generalizability of the findings to other populations or healthcare settings. However, because of the retrospective case–control design, reliance on FFQ data, and potential recall bias or diet changes around diagnosis, these findings should be interpreted as associations rather than proof of causality. Furthermore, despite adjusting for several variables, residual confounding from unmeasured factors such as genetic predisposition or environmental exposures cannot be ruled out.

## Conclusion

A high diet quality, as indicated by higher total GDQS and total PDQS scores, was associated with lower odds of breast cancer in this hospital-based case–control study. These findings highlight a potential role of overall diet quality in breast cancer etiology but should be interpreted as observational associations rather than evidence of causal or therapeutic effects.

## Data Availability

The original contributions presented in the study are included in the article/supplementary material, further inquiries can be directed to the corresponding author.
